# Predictive Factors for Medical Consultation for Sore Throat in Adults with Recurrent Pharyngotonsillitis

**DOI:** 10.1155/2016/6095689

**Published:** 2016-04-28

**Authors:** T. Koskenkorva, P. Koivunen, O.-P. Alho

**Affiliations:** ^1^Department of Otorhinolaryngology and Head and Neck Surgery, Oulu University Hospital, Finland; ^2^PEDEGO Research Unit, University of Oulu, P.O. Box 5000, 90014 Oulu, Finland; ^3^Medical Research Center Oulu, Finland

## Abstract

*Objects*. To seek patient- and episode-related factors that associate with medical consultation for acute sore throat because these factors may affect the patient being referred to specialist care and tonsillectomy for recurrent pharyngotonsillitis.* Methods*. In a secondary analysis of two prior randomised controlled trials, sore throat episodes and medical visits were explored among 156 adult patients referred for tonsillectomy because of recurrent pharyngotonsillitis.* Results*. The 156 patients (104 females, mean age of 26 years) suffered from 208 acute pharyngotonsillitis episodes during 5-6 months of follow-up. Forty (25%) patients visited a physician, and female gender (adjusted hazard ratio, HR, 3.3; 95% confidence interval 1.4–8.0) and finding of chronically infected tonsils (HR 2.7; 1.2–6.1) were associated with medical consultation. Thirty-six (17%) episodes led to medical consultation during the first 7 days of symptoms. Presence of severe throat pain was related to medical visit (HR 4.3; 1.0–18.5).* Conclusions*. Even among patients with recurrent pharyngotonsillitis, the acute sore throat episodes were usually mild and only few resulted in medical consultation, with female gender, chronically infected tonsils, and having severe throat pain increasing the consultation rate.

## 1. Introduction

Recurrent pharyngotonsillitis is a common health problem causing medical appointments, antimicrobial treatments, and missed workdays or schooldays [1]. Previously, we conducted two randomised controlled trials in adults with recurrent pharyngotonsillitis on the efficacy of tonsillectomy [2, 3]. While we noticed that tonsillectomy reduced the number of acute pharyngotonsillitis episodes and increased the quality of life, we also found out that the patients graded most of their episodes as nonsevere [3, 4]. Thus, it seems like something other than the severity of throat pain may affect whether or not the patient consults the health care system during a pharyngitis episode.

Here, we sought patient- and episode-related factors that would explain whether or not the patient made a medical visit for acute sore throat both before and after tonsillectomy.

## 2. Materials and Methods

Study design was a secondary analysis of two randomised controlled trials exploring the effect of tonsillectomy in reducing further episodes of sore throat in adult patients with recurrent pharyngotonsillitis. These trials have been described previously [2, 3]. In brief, the first trial (streptococcal material) included 70 adults who had had at least 3 episodes of pharyngotonsillitis in 6 months or 4 episodes in 12 months, with one of them having to be of streptococcal origin. The second trial (pharyngotonsillitis material) involved 86 adults who had had at least 3 episodes of pharyngotonsillitis of any origin in 12 months. Both trials were registered in ClinicalTrials.gov (NCT00136877 and NCT00547391). Unpublished preliminary results are presented in doctoral thesis [5].

Half of the participants were randomly assigned to immediate tonsillectomy and the other half to a waiting list. Thus, we followed up both operated and unoperated patients for approximately 5 to 6 months. We collected background data and examined the patients. Appearance of the palatine tonsils was evaluated by the study physician. Grades 3 and 4 tonsils according to the grading scale by Brodsky were regarded as large [6]. Tonsils were regarded as chronically infected if they were inflamed and cryptic and these crypts contained debris. Tonsils were defined as scarred if they were harder than usual when palpated. The patients were given a symptom diary where they recorded the presence of throat pain, fever, cough, and rhinitis. In the pharyngotonsillitis material, also the severity (mild, moderate, or severe) of the throat pain was recorded. The patients were told to seek medical advice for their acute symptoms during the trial in exactly the same way they had done before. Physicians recorded the date, location, diagnosis, and treatment of acute episodes as guided by trial instructions. Acute pharyngitis is a sudden inflammation or infection of the pharynx, usually causing a sore throat. Acute tonsillitis, on the other hand, is a sudden infection or inflammation of one or both of the palatine tonsils. In this paper, we use the term pharyngotonsillitis for simplification for all the patients, since we have studied patients with sore throat episodes both before and after tonsillectomy. We considered at least two consecutive days with a sore throat as an episode.

For descriptive data, we calculated means with standard deviations or ranges. To explore patient-related variables and medical consultations, we constructed Kaplan-Meier curves for each of the 156 patients, starting from the date of the randomisation. The primary end point was the first visit to a physician because of pharyngotonsillitis. We calculated the cumulative incidence of a medical consultation up to 5 months of follow-up using life-table analysis. We used a Cox proportional-hazards regression model to explore associations between various patient-related factors and medical consultations for sore throat. The cumulative risk of visiting a physician was calculated as an adjusted hazard ratio (HR) with 95% confidence intervals (CIs). The model included the following variables: age (≤20 or >30 years versus 21–30 years); gender; tobacco use; prior streptococcal pharyngotonsillitis; number of episodes of pharyngotonsillitis in the prior 6 months (≤3 versus >3); and chronically infected tonsils. The associations between patient-related factors and medical consultations were similar in the episodes before and after tonsillectomy, so these two datasets were combined.

To study episode-related factors and medical consultations, we similarly constructed Kaplan-Meier curves of each of the 208 episodes of acute pharyngotonsillitis, starting from symptom onset. The primary end point was the first visit to a physician because of pharyngotonsillitis during the first 7 days. We calculated the cumulative incidence of a medical consultation up to the first week of symptoms using life-table analysis. We used a Cox regression model to explore the associations between various episode-related factors and medical consultations. The cumulative risk of visiting a physician was calculated as an adjusted HR with 95% CIs. The model included the following variables: maximum throat pain during the episode (mild versus moderate versus severe); fever; other respiratory symptoms; and pre- versus postoperative episode.

## 3. Results 

**Figure 1:**
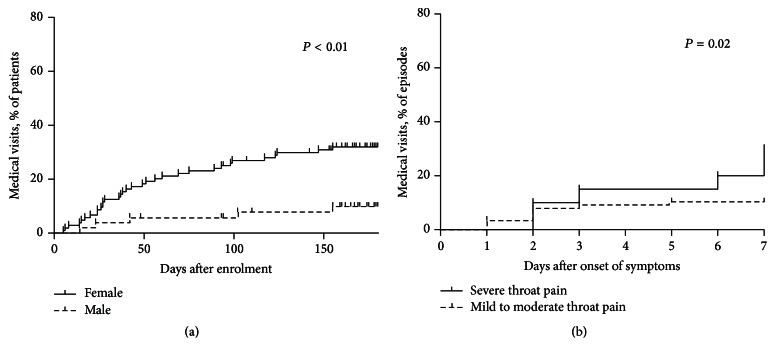
(a) Proportion of adult patients with recurrent pharyngotonsillitis who visited physician for acute pharyngotonsillitis during a follow-up of 6 months according to sex (*N* = 156 patients, differences between groups tested with log rank test). (b) Proportion of acute pharyngotonsillitis episodes that led to medical consultation during the first 7 days according to whether the patient had had severe throat pain (*N* = 135 episodes, where data on severity of throat pain was available, differences between groups tested with log rank test).

Baseline characteristics of the patients are shown in Table 1. The mean length of the follow-up was 5.9 (SD 1.2) months. Forty (25%) of the 156 patients visited a physician for acute pharyngotonsillitis. Female gender (HR 3.3, 95% CI 1.4–8.0) and chronically infected tonsils at clinical examination (HR 2.7, 95% CI 1.2–6.1) were factors that associated with medical consultation during a pharyngotonsillitis episode (Table 2) (Figure 1(a)). Age, tobacco use, prior streptococcal pharyngotonsillitis, and frequent pharyngotonsillitis episodes did not affect the consultation rate.

Seventy-eight patients had 208 acute pharyngotonsillitis episodes (mean duration of 6.2 days, SD 4.5). Thirty-six episodes (17%) led to a medical consultation during the first 7 days of symptoms and the mean duration of symptoms before consultation was 4.1 (SD 3.6) days. The risk for medical visit increased after the third day of symptom onset (Figure 1(b)). Only the fact that the patient had had severe throat pain during the first 7 days correlated significantly with the medical consultation (HR 4.3, 95% CI 1.0–18.5) (Table 2). In contrast, having had moderate throat pain, fever, the presence of other respiratory symptoms, and whether the episode was pre- or postoperative were not associated with the medical consultation.

## 4. Discussion

We found that acute pharyngotonsillitis episodes both before and after tonsillectomy lasted less than a week; on average, majority of cases involved at most only nonsevere throat pain and less than one-third involved fever. Thus, it was not surprising that only one of every six episodes led to medical consultation.

We found that the medical appointment rate for acute pharyngotonsillitis was higher in women than in men. This higher consultation rate may lead to an increased amount of recurrent pharyngotonsillitis diagnoses and may further increase referrals to specialist care for tonsillectomy among women. In fact, two-thirds of the patients in our study population—being secondary care referrals—were female, supporting this assumption. Earlier literature reports that women have higher consultation rates with specialists for consideration of paranasal surgery due to recurrent acute rhinosinusitis, as well [7–9].

Severe throat pain during the acute pharyngotonsillitis episode increased the probability of a medical visit after the third day of symptom onset. This finding indicates that patients are mainly looking for relief from severe pain and also suggests that patients may be aware that milder episodes usually heal by themselves without medical care [10, 11].

In contrast, such factors as age, tobacco use, streptococcal pharyngotonsillitis in history, fever, and whether the episode occurred before or after tonsillectomy did not correlate with a medical visit.

To minimize the effect of participating in a trial, we emphasized that it was important that the patients seek medical advice for their symptoms during the trial in exactly the same way they had done before. This study was done in a country where national guidelines suggest that tests should be done for *β*-haemolytic* Streptococcus* A when treating acute pharyngotonsillitis, which must be considered when the generalizability of the results is evaluated [12].

To conclude, even among adult patients who suffered from recurrent pharyngotonsillitis episodes, the overwhelming majority of the acute pharyngotonsillitis episodes were mild and only few resulted in a medical consultation, with female gender, chronically infected tonsils, and having severe throat pain increasing the consultation rate.

## Figures and Tables

**Table 1:** Demographic and baseline characteristics of adult patients with recurrent pharyngotonsillitis and features of pharyngotonsillitis episodes during follow-up (mean of 5.9 (SD 1.2)^*∗*^ months). Figures are numbers (percentages) unless otherwise indicated.

Characteristic/feature	
Patient-related (*N* = 156 patients)	
Mean (range) age (years)	26 (14–65)
Female	104 (67)
Tobacco use	60 (39)
History of allergy	50 (32)
Risk factors for pharyngotonsillitis:	
More than four people in family	35 (23)
Use of same toothbrush > 3 months	36 (23)
Mean (SD)^*∗*^ number of previous episodes of acute	
pharyngotonsillitis diagnosed by physician:	
During past 6 months	3.3 (1.4)
During past 12 months	5.0 (2.0)
Group A *Streptococcus* pharyngotonsillitis diagnosed by physician in the past	130 (83)
Frequent throat pain	54 (35)
Tonsils at baseline according to clinical assessment^†^:	
Large	48 (31)
Chronically infected	18 (12)
Scarred	104 (67)

Episode-related (*N* = 208 acute pharyngitis episodes)	
Preoperative	161 (77)
Postoperative	47 (23)
Involved besides throat pain	
Other respiratory symptoms	115 (55)
Fever	61 (29)
Severity of throat pain at most during episode	
Mild	54 (40)^‡^
Moderate	60 (44)^‡^
Severe	21 (16)^‡^

^*∗*^SD: standard deviation. ^†^More than one clinical feature possible. ^‡^Data available for 135 episodes.

**Table 2:** Association between various patient- and episode-related factors and having a medical consultation during an episode of acute pharyngotonsillitis in a material based on two randomised trials in Finland [2, 3].

Characteristic		Hazard ratio^**∗**^	95% Confidence interval^**∗**^	*P*
Patient-related (*N* = 156 patients)				
Age	≤20 or >30 years	1.0		0.40
	21–30 years	0.7	0.4–1.5	
Sex	Male	1.0		<0.01
	Female	3.3	1.4–8.0	
Tobacco use	No	1.0		0.17
	Yes	0.6	0.3–1.2	
Prior streptococcal	No	1.0		0.51
pharyngotonsillitis	Yes	1.3	0.6–3.3	
Frequent episodes of	No	1.0		0.59
pharyngotonsillitis^*∗∗*^	Yes	1.2	0.6–2.3	
Chronically infected	No	1.0		0.01
tonsils^†^	Yes	2.7	1.2–6.1	

Episode-related (*N* = 208 acute pharyngotonsillitis episodes)				
Postoperative episode		1.0		0.62
Preoperative episode		1.5	0.3–6.7	
Maximum throat pain^‡^	Mild	1.0		
	Moderate	1.6	0.5–5.8	0.44
	Severe	4.3	1.0–18.6	0.05
Fever	No	1.0		0.62
	Yes	0.7	0.2–2.3	
Other respiratory	No	1.0		0.83
symptoms	Yes	1.1	0.4–3.1	

^*∗*^Cox regression model.

^*∗∗*^>3 episodes in 6 months.

^†^Found at clinical examination at enrolment.

^‡^Calculated among those episodes, where data on severity of throat pain was available (*N* = 135).
